# Internode elongation and strobili production of *Humulus lupulus* cultivars in response to local strain sensing

**DOI:** 10.1038/s41598-021-88720-8

**Published:** 2021-04-27

**Authors:** William L. Bauerle

**Affiliations:** Department of Horticulture and Landscape Architecture, Graduate Degree Program in Ecology, Colorado State University, Fort Collins, CO 80523 USA

**Keywords:** Agroecology, Agroecology, Ecophysiology, Biophysics, Ecology, Ecology, Environmental sciences

## Abstract

Three different cultivars of *Humulus lupulus* L. were subjected to a regime of internode touch and bending under greenhouse conditions. Experiments were performed to assess intraspecific variability in plant mechanosensing, flower quality, and yield to quantify the thigmomorphogenic impact on plant compactness and flowering performance. Touching and/or touching plus bending the plant shoot internodes located in the apical meristem zone decreased internode elongation and increased width. The growth responses were due partly to touching and/or touching plus bending perturbation, 25.6% and 28% respectively. Growth of new tissue within the local apical portion of the bine continued to remain mechanosensitive. The number of nodes and female flowers produced was unaffected by either type of mechanical stress. The study provides evidence that thigmomorphogenic cues can be used as a hop crop management tool to increase bine compactness and increase node density per unit area. The findings have broad implications for hop production; production can more readily take place in a confined greenhouse space with the aid of mechanical stimulation to control plant growth without sacrificing yield or flower quality.

## Introduction

The yield potential of *Humulus lupulus* L. (hop) flowers is linked to the quantity of fertile nodes developed prior to and during the seasonal photoperiod shift that transitions the plant from the vegetative to generative phase of the life cycle. Given that the yield of hops are heavily dependent on the number of fertile nodes per plant, a grower would need to accommodate as many hop nodes per plant as possible to maximize production in a given area. If environmental resources are not limiting (such as in controlled environment production that provides supplemental plant resources e.g. CO_2_ and light), novel approaches to increase the node count and total flower yield of hops would be beneficial to production e.g.^1^.

Mechanical perturbation, including a subtle touch, is a known stimuli in some plant species. Depending on the species, mechanical stimuli can trigger changes in phytohormones, gene expression, morphology, pithiness, flowering time, growth rate, senescence and stomatal aperture e.g.^2^. Jaffe (1973) termed this growth response ‘thigmomorphogenesis’ (*thigma* means touch in Greek) where the most common response is a decrease in length and an increase in radial expansion^3,4^. With the exception of a few plant species (e.g. *Mimosa pudica* and *Dionaea muscipula*), plants have a slow touch response time^5^. Within an individual plant, the youngest tissue is the most sensitive to touch and it will show the largest magnitude of response to mechanical perturbation^2^. In hop bines, the youngest plant tissue is located at the terminal apical meristem and progresses in age toward the root system. Once laterals develop from the main shoot, a similar age structure persists from the lateral tip toward the main bine. In addition, hops naturally rotate their apical meristem in a circular diameter of approximately 0.5 m in search of a support structure^6^. Once a structure is encountered, the bine circles the support clockwise and silica hairs on the bines surface help it adhere to the support. Thus, the growth and development ontogeny characteristics of hops make the species an ideal candidate for purposeful mechanical perturbation of the apical meristem. Nevertheless, the author knows of no published data to substantiate or refute whether hops demonstrate a thigmomorphogenic response induced by “artificial” mechanical signals.

Hop production, whether it take place outdoors or within a controlled environment confined space, presents a challenge imposed primarily by the plant’s physical size (length). In comparison with small stature species, hop bines require a certain node count to flower. This is due to a protracted variety-specific juvenile phase during which they are incapable of flowering unless 12–25 nodes are visible to the naked eye^7^. The juvenile-adult transformation stipulation results in non self-supporting bines that are of significant length and morphological complexity, making them difficult subjects for experimental work or production in a confined space.

By and large, internode length determines a plants compactness. In hops, it determines total bine length and node quantity per unit area. Recently, hop breeding efforts have focused on low trellis (dwarf) varieties e.g.^8–10^. Knowledge regarding hop’s thigmomorphogenic responses would be useful for managing internode length and node development per unit area. Furthermore, in conjunction with the recent finding that hops lack a vernalization requirement^11^, expertise in this area would complement speed breeding by benefiting growers working in a restricted production area. For example, the ability to grow compact hop plants with a nigh node density in a controlled environment space may be of value to off-season hop production. The objective of this study was to investigate the sensitivity of hops to a bending and touch stimulus under conditions approximating commercial greenhouse production. A factorial experiment was set up to assess the response of hop node count and development, internode length, and flower yield and quality (with and without exposure to touch and/or touch plus internode bending). We hypothesized that the density of nodes per unit bine length could be increased via mechanical perturbation. In doing so, we quantified the thigmomorphogenic influence on flowering performance in a confined space greenhouse setting while minimizing potential confounding effects of gravity, self-weight, phototropism, and wind. Differences in cultivar responses to touch and stem bending are also discussed.

## Materials and methods

### Environmental conditions

Primary measurements occurred during 2017–2018 in the Horticulture Center at Colorado State University, Fort Collins, CO, where greenhouses allowed for wind-protected enclosed studies to eliminate periodic wind gusts (a potential plant thigmomorphogenic stimuli). Greenhouse conditions were programmed to a set point air temperature of 26 °C during photoperiod and 20 °C during the dark with a 45 min temperature step change between the two, 50% relative humidity (RH; %), and supplemental photosynthetically active radiation (PAR) of 100—700 umol m^−2^ s^−1^ during the photoperiod (Philips lighting, Amsterdam, The Netherlands). Controllers were programmed to permit the maximum amount of light penetration (shade cloth was only pulled when intense solar radiation and temperatures demanded additional cooling efforts) where daytime ambient PAR at the canopy surface was generally 800–1,100 µmol m^−2^ s^−1^. Daytime temperatures over the experimental period averaged 26.4 °C, but in some instances temperatures climbed higher despite continuous cooling. Supplemental humidity was provided via an evaporative cooling pad, and the daytime saturation vapour pressure deficit (VPD) averaged 1.9 kPa. Air temperature and RH were measured using EHT RH/temperature sensors and PAR using AQO-S PAR photon flux sensors mounted at the top of the canopy (Meter Devices, Pullman, WA, USA).

### Plant material

Over the course of the study, female varieties of tissue culture propagated plantlets were used (Summit Plant Labs Inc., Fort Collins, CO, USA). The varieties ‘Cascade’, ‘Cashmere’, and ‘Centennial’ were selected from public cultivars. Their origin, indicative harvest time, and brewing use are reported in Supplementary Table S1. In an initial experiment, in order not to confound juvenility with flowering potential and node number among crop cycles, the minimum number of nodes for flower induction was quantified along a gradient in plant sizes and visible node development. In so doing, plants of progressively greater node development were quantified from the base of the plant to the last visible node prior to the apical meristem. It was determined that all cultivars in the study are ‘ripe to flower’ when ≥ 20 nodes are visible to the eye.

Plantlets were transplanted into 11 L bato buckets containing 100% horticulture grade perlite and placed under supplemental PAR and an extended photoperiod to control day length at 18 h (Philips LED lighting, Amsterdam, The Netherlands). Containers were spaced 0.61 m (within rows) in 20 m rows per cultivar with 1.52 m between rows. One bine per container was initially grown vertically under Earth’s gravity. Thus, bines in this study received 0.93 m^2^ of space per bine. Bines were trained to a string trellis at approximately 0.5 m of initial bine length and all bines were grown at 90° on vertical white polypropylene twine until 20 visible nodes developed. We note that although there is not a ‘standard’ bine spacing per unit area for hops, the plant density in this study equated to 10,764 bines per ha^−1^, which is a plant density similar to manual versus mechanized field hop production^6^.

An automated irrigation system supplied ample nutrient and water conditions by feeding with a complete fertilizer (15-2-24, Aagrozz Inc., Wooster, OH, USA) via pressure compensating drip emitters (ML Irrigation Inc., Laurens, SC, USA). Initially, all pots were watered to saturation and permitted to drain for 18 h and thereafter container capacity was maintained daily. White plastic sheeting was cut and placed on the substrate surface to eliminate evaporation. Photoperiod was reduced to 14 h for flower induction once 35 nodes were visible to the naked eye and black out blinds were used to prevent light pollution.

### Treatments

In hops, only the four apical internodes are able to elongate^12^. We conducted an initial experiment to verify that hops possess a thigmotropic response as opposed to a phototropic response with respect to their direction of ascent. To do so we mounted twelve 350 W Philips metal halide lights (Philips lighting, Amsterdam, The Netherlands) horizontally adjacent to hop rows and verified (a) that the hop apical meristem autonomously grows vertically in a right handed clockwise helix, and (b) once it senses (touches) a support structure it autonomously wraps around the structure for physical support as it climbs. We pre-set trellis twine at slopes of 90, 75, 60, 45, and 30°’s and found that once the bine apical meristem came in contact with the trellis twine it proceeded to naturally climb the twine in a clockwise rotation, adhering to the twine via stiff silica hairs that act as hooks (Supplementary Fig. S1). Once it wrapped itself clockwise around the trellis support structure, it proceeded to climb the twine at the pre-set twine slopes regardless of the horizontally adjacent light illumination. Moreover, adjacent parallel PAR ≥ 450 umol m^−2^ s^−1^ above that of incoming overhead PAR did not deter apical meristem vertical ascent. However, we note that twine slopes less than 45° to the vertical would occasionally exceed the natural helix radii of the hop bines thigmotropic sensing apical meristem, causing the downward hanging bine to wrap in a clockwise helix back onto itself in search of a vertically oriented physical support. This may possibly indicate an additional gravitropic response versus a phototropic response within the hop apical meristem.

**Figure 1:**
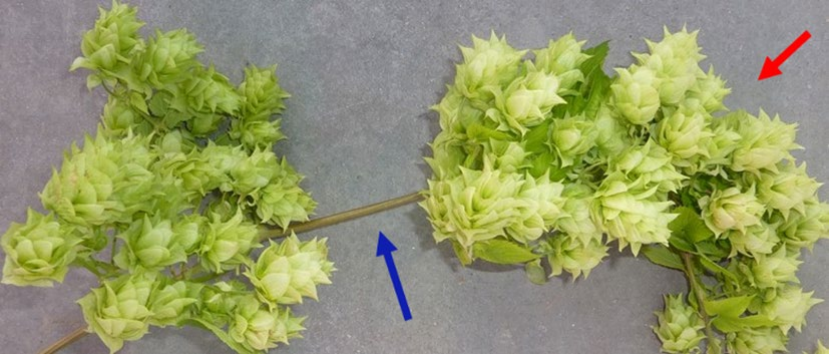
67 days into the crop cycle a representative example of cultivar ‘Centennial’ hop cone production on a node where the internode was not previously bent (blue arrow and left side of photo) versus a subsequent node that was bent at 90° (red arrow and right side of photo). Moreover, the red arrow indicates a shorter internode that results in a higher density of cones along the length of the bine (90° bending), whereas to the left of the blue arrow indicates a sparser cone density sans bending.

**Figure 2:**
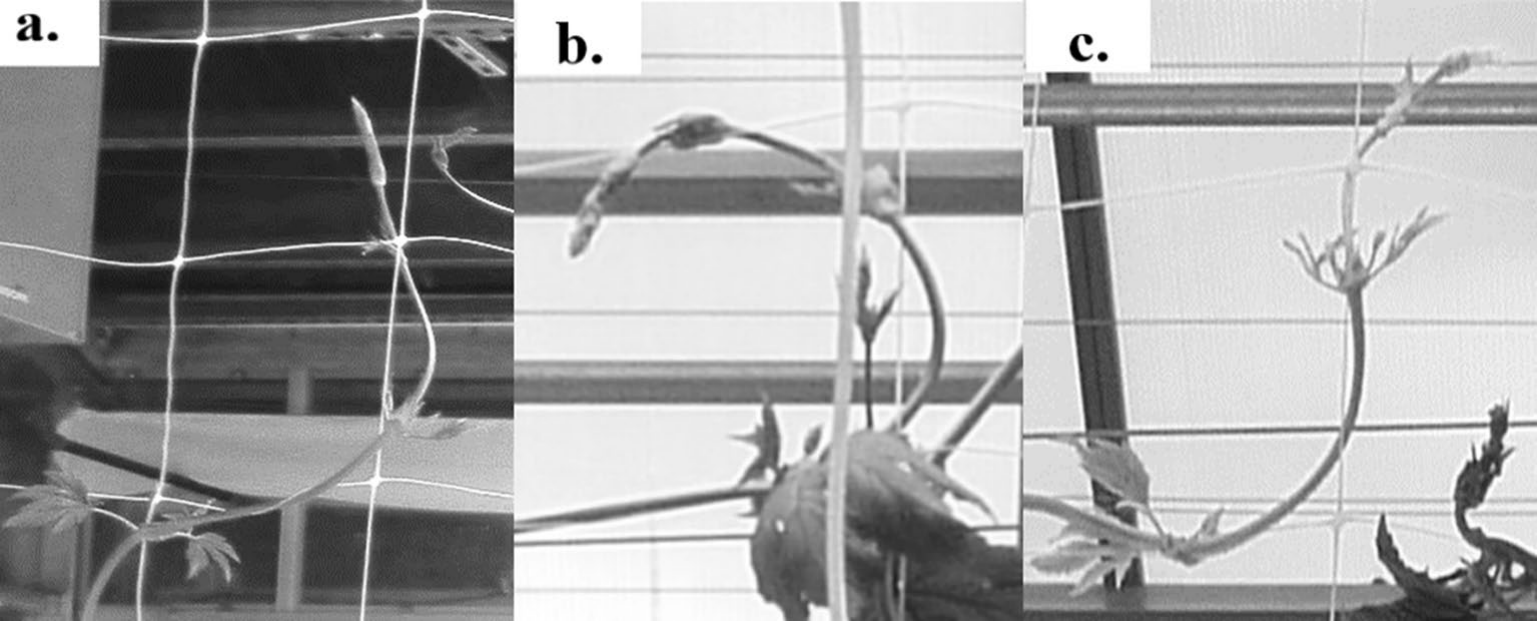
The 24 h sequence of forming a 90° bend in the bine between the second and third visible node from the apical meristem (**a**) first the apical meristem is bent at approximately 90°, (**b**) secondly it is undisturbed for 24 h of growth, and (**c**) thirdly the second and third visible node from the apical meristem is artificially bent at 90° and repositioned clockwise around the netting so that it can proceed to grow up the 15.25 cm rise then run of netting (e.g. a 15.25 cm × 15.25 cm rise and run is sequentially bent into the shape of a bine staircase at an average slope of 45°). Note, images are black and white for color consistency to not distract the reader by the color variations brought about by the red and blue LED illumination under daytime versus nighttime conditions.

**Figure 3:**
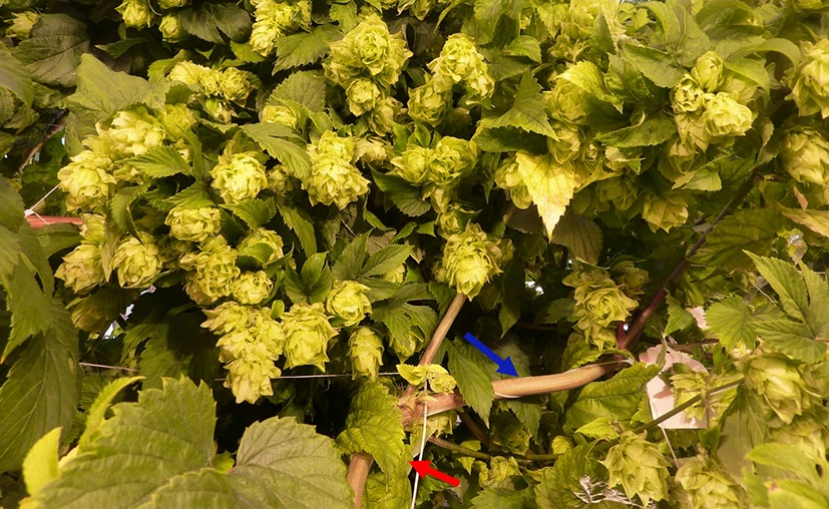
Representative example of cultivar ‘Centennial’ hop cone production within a subsection of crown guided through netting (67 days into the crop cycle). The red arrow indicates the local internode strain location at the junction of the netting 15.25 cm vertical rise transition to the 15.25 cm run and the blue arrow indicates the subsequent 15.25 cm horizontal run.

Six treatments were applied, each treatment to six independent bines per cultivar (n = 6). Once 20 visible nodes were present, the bines were lowered by lengthening the string in the vertical direction which permitted the plants to be lowered and the bine coiled around the container in order to locate node 20 at a uniform height of 0.9 m among all plants (approximately 3 m of initial bine length). The treatments were applied from node 20 through node 40 and consisted of: (1) the control treatment free to climb a 90° vertical white polypropylene twine (FC), (2) the twine versus netting treatment free to climb a strand of 90° white polypropylene vertical netting (FN), (3) the gravitropic and/or slope response treatment free to climb the twine set at a 45° slope (F45), (4) a periodic touch treatment with twine set at a 45° slope and internode touching stimuli applied once every 24 h at the midpoint of the second and third true node from the apical meristem (T45) (location pointed to by blue arrow in Fig. 1), (5) the twine versus netting periodic touch treatment where a single strand of trellis netting was set at a 45° slope and internode touching stimuli was as in treatment four (N45), and (6) the internode bending treatment were bines were trained to the netting at a mean 45° slope (B90) (Figs. 2, 3). Specifically, the B90 treatment consisted of internode touch as in T45 and N45 with the addition of mechanical perturbation placed at the midpoint of the second and third true internode from the apical meristem to form an approximate 90° bend in the bine (performed in conjunction with touch once every 24 h). The bent state of treatment B90 was an approximately 90° bend small displacement of the bine in a clockwise rotation around the netting resulting in a internode bend once every 15.25 cm of netting rise and run, i.e. the natural clockwise bine rotation along with guiding the bine along the 15.25 cm × 15.25 cm netting grid structure (Fig. 2a–c). The netting secured the internode bend in place where Fig. 2 illustrates the bending sequence as the bine grew over a 24 h period. Implementing the bend takes approximately 5–10 s^−1^ per bine per 24 h and the application duration equates to ~ 30 days out of the 90—120 day crop cycle (~ 150–300 s^−1^ per bine per crop cycle). Note, touch in treatment T45 was applied at a 45° slope instead of 90° to increase the overall bine length between the ground surface and the bine terminal tip.

### Touching and bending treatment application

The bending and/or touching stimulus was applied once per 24 h to T45, N45, and B90 bines between nodes 20 to 40 after which bine touching and bending ceased. The touching stimulus in T45 and N45 consisted of holding the internode (located between the second and third node from the apical meristem) between the thumb and forefinger and wrapping clockwise along a 45° slope. For the B90 treatment, bine touch procedures were identical to T45 and N45 with the addition of wrapping the bine internode at 90° via a perpendicular 15.25 cm rise and run (15.25 × 15.25 cm netting grid dimensions). Once the bine was placed in contact with the twine and/or netting, the bine would naturally adhere to the lattice via downward facing silica hooks (e.g. Supplementary Fig. 1). We note that local bending deflections were the primary target of this study due to the flexible nature of the younger part of hop bines. Specifically, the bending stiffness of hop bines changes lengthwise along the stem with the most flexible portion closest to the apical meristem. This hop bine growth and development aspect together with the netting grid dimensions allowed for a local strain-based deformation of the longitudinal stem. Thus, treatment B90 imposed a commercially applicable tissue strain at a standardized location (similar rigidity) within the flexible bine elongation zone (Fig. 2a–c). The hands-on methodology retains commercial applicability, yet permits the bending force to be almost proportional to the sum of strains^13^. Moreover, the local internode bending took place within the zone of competent mechanoperception tissue i.e. the local stem portion with the youngest tissue that would perceive the highest mechanosensing strain signal for growth reduction e.g.^13,14^. Treatments FT, FN, and F45 were allowed to grow free of mechanical stimulation (untouched) as hop bines autonomously rotate in a right-handed clockwise helix. The 45° twine angle of F45 and T45, i.e. sans bending, allowed separation from a possible influence of gravitropism due to the lack of touch stimuli as a mechanosensing stimuli can be triggered with as little as 0.3 mg of perturbation^15^.

### Bine growth and yield

Six plants (n = 6) of each cultivar per treatment were periodically sampled for internode elongation and width between node 20 – 40. The six plants were treated as replicates in the analyses and the remaining plants per cultivar within the rows were randomly dispersed among the sampled plants to act as buffers. At harvest, the internode length and width from node 20–40 was measured and nodes 20–40 manually picked. Fresh cone weights were measured on a per replicate basis and six random 100 g cone subsamples within node 20–40 were taken from each cultivar at harvest for dry matter content. The subsamples were weighed fresh and then immediately dried at 45 °C in forced air until reaching approximately 9% moisture. The samples were then reweighed, vacuum-sealed in clear plastic bags, and stored at 2 °C for determination of chemical constituents. Cone yield per bine replicate from node 20–40 was calculated for each cultivar.

### Cone acid analysis

Contents of α and β-acids were determined by the spectrophotometric technique of the American Society of Brewing Chemists^16^. Cones of each dried sample were ground to a fine powder per cultivar and a homogenized sample was extracted from the lot of dried raw hops; 2.5 g of dried hop powder was weighed to the nearest mg and placed in a 100 mL beaker with 50.0 mL of methanol. The aliquot was stirred for 30 min at room temperature and the extract was then force filtered via centrifuge to remove particulate matter. A 50 μL aliquot of the filtrate was placed in a 25 mL volumetric flask and the flask was then filled with methanolic NaOH (0.5 mL of 6 M NaOH in 250 mL of methanol). An aliquot of this solution was placed in a 1 cm quartz cell and its absorbance values obtained for the wavelengths of 275, 325, and 355 nm against a blank of 50 μL of methanol in 25 mL of methanolic NaOH (Hach 6000 spectrophotometer; Loveland, CO, USA).

### Statistical analysis

The experimental design was a balanced full factorial (each tracked plant per treatment was an experimental unit treated as a replicate (n = 6)). Two way analysis of variance (ANOVA) and two tailed t-tests were used to analyze the significance of internode elongation, width, yield, and cone quantity among treatments (SPSS; IBM Analytics, www.ibm.com/analytics/, USA). Differences between means were considered significant when the *P* value of the ANOVA *F* test or the t-value was < 0.05.

## Results and discussion

**Figure 4:**
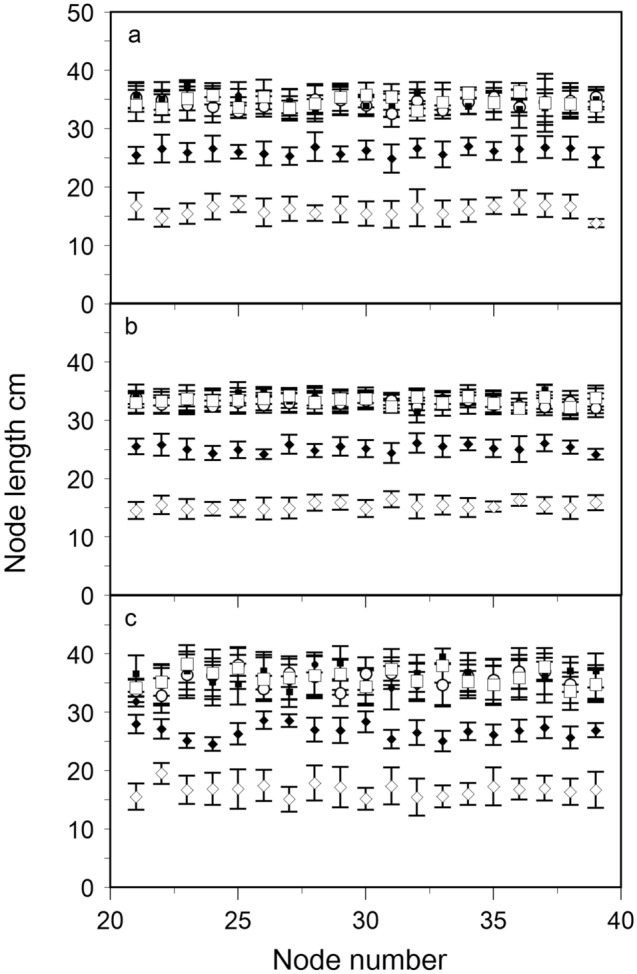
The kinetics of bine elongation from node 20–40 + /- standard deviation for (**a**), cultivar ‘Cascade’, (**b**), cultivar ‘Centennial’ and (**c**), cultivar ‘Cashmere’ control free to climb at 90° (FC; ●), free to climb a strand of 90° netting (FN; ○), free to climb twine at 45° (F45; ■), touch on twine at 45° (T45; □), touch on a strand of trellis netting at 45° slope (N45; ◆), and 90° internode bending on netting at a 45° mean slope (B90; ◇). Vertical bars represent standard deviations of six replicates (n = 6). Internode lengths among treatments are statistically different from each other (*P* < 0.01).

Figure 4 illustrates the length of fertile internodes 20–40 within the various treatments of: FC, FN, F45, T45, N45, and B90. The FC, FN, and F45 treatments grew lengthier internodes from node 20–40 than the T45, N45, and B45 treatments (Fig. 4). Internode width, however, was greater in T45, N45, and B90 as compared to the undisturbed FC, FN, and F45 treatments (Table 1). The T45 and N45 treatments had a 27.9% and 26.6% reduction in internode elongation compared to the FC, FN, and F45 treatments. Of the treatments, B90 had the shortest internodes and widest internode thickness between node 20–40 (Tables 1, 2). Due to the shorter internode lengths in the mechanically affected treatments, the density of nodes per unit area was ~ 25% greater in T45 and N45 from node 20–40 and an additional 28% shorter in B90. In other words, B90 internodes were ~ 54% shorter between nodes 20–40 as compared to the untouched treatments and had the densest node concentration (cf Fig. 4 and Table 2). Both touched and bent bines were significantly reduced in elongation (Table 1; P < 0.01 and P < 0.01 respectively). We note that although interspecific thigmomorphogenic variation has been shown in rainforest tree species^17^, the intraspecific internode variation within this study was not significant (Table 2). Regarding internode width, only cultivar ‘Centennial’ was significantly different from ‘Cascade’ and ‘Cashmere’ (Table 1). This may partly be due to the shared genetics of the cultivars in this study where ‘Cascade’ and Centennial’ share ‘Fuggle’ parentage and ‘Cascade’ is a parent of ‘Cashmere’.

**Table 1:** Mean internode width (mm) of main bine internodes between node 20 and 40. Control free to climb at 90° (FC), free to climb a strand of 90° netting (FN), free to climb twine at 45° (F45), touch on twine at 45° slope (T45), touch on a single strand of trellis netting at 45° slope (N45), and 90° internode bending on netting at a 45° mean slope (B90). Standard deviation terms are reported as standard deviation of the difference of the means for cultivars ‘Cascade’ (n = 6), ‘Centennial’ (n = 6), and ‘Cashmere’ (n = 6). Different letters indicate significant difference among treatments (*P* < 0.05).

Cultivar	FC	FN	F45	T45	N45	B90
*‘Cascade’*	7.34 ± 0.08^a^	7.34 ± 0.09^a^	7.33 ± 0.10^a^	8.36 ± 0.07^c^	8.38 ± 0.07^c^	10.21 ± 0.15^d^
*‘Cashmere’*	7.28 ± 0.07^a^	7.27 ± 0.07^a^	7.29 ± 0.08^a^	8.43 ± 0.08^c^	8.33 ± 0.06^c^	10.17 ± 0.14^d^
*‘Centennial’*	7.81 ± 0.10^b^	7.79 ± 0.11^b^	7.83 ± 0.08^b^	8.50 ± 0.09^c^	8.52 ± 0.09^c^	10.23 ± 0.16^d^

**Table 2:** Mean internode length (cm) of main bine internodes between node 20 and 40. Control free to climb twine at 90° (FC), free to climb netting at 90° netting (FN), free to climb twine at 45° (F45), touch stimuli on twine at 45° slope (T45), touch stimuli on netting at 45° slope (N45), and touch stimuli plus 90° internode bending on netting at a 45° mean slope (B90). Standard deviation terms are reported as standard deviation of the difference of the means for replicates of cultivars ‘Cascade’ (n = 6), ‘Centennial’ (n = 6), and ‘Cashmere’ (n = 6). Different letters indicate significant differences among treatments (*P* < 0.05).

Cultivar	FC	FN	F45	T45	N45	B90
*‘Cascade’*	34.5 ± 2.1^a^	34.3 ± 2.0^a^	34.7 ± 2.3^a^	25.2 ± 2.2^b^	26.1 ± 1.8^b^	16.0 ± 2.1^c^
*‘Cashmere’*	33.3 ± 1.5^a^	32.9 ± 1.5^a^	33.4 ± 1.8^a^	24.3 ± 1.6^b^	25.2 ± 1.5^b^	15.3 ± 1.5^c^
*‘Centennial’*	35.4 ± 3.1^a^	35.5 ± 2.9^a^	36.2 ± 3.4^a^	25.1 ± 3.1^b^	26.7 ± 1.9^b^	16.6 ± 2.6^c^

One of the main criteria for controlled environment production in a confined space is compact plant growth. As compared to ornamental crop production where the focus is on visual quality, efficient production of edible flower crops focuses on flower quality and yield per given cultivated area. For hop production specifically, the primary goal of mechanostimulation is to control internode length under confined greenhouse space conditions without causing a reduction in the node development rate, bine yield, or flower quality^18^. Chemical growth regulators are one means to control plant compactness, but their toxicity to human health could potentially be carried into the hop strobili acids and oils, a primary beer ingredient e.g.^19^. Being that hop flowers are consumed in beer as a flavor and bitterness ingredient, we deployed mechanical perturbation as an alternative to potentially toxic synthetic growth substances. Although internode length decreased with touch, the combination of touching and bending approximately doubled the decrease in internode length in all three cultivars studied (Table 2). One could argue that the response is attributable to an “observer” effect^20^. However, treatments T45 and N45 allowed us to tease out the potential for an “observer” effect from B90 by maintaining an identical touch stimuli among T45, N45, and B90. Furthermore, to minimize variation in the pressure applied via touch, the stimuli was consistently implemented by the same observer^21^. Thus, variation in internode lengths and widths within a cultivar and treatment was minimal; possibly a result of a consistent observer, alternatively the response we observed in hops was in the form of a gradual morphogenetic response resulting in a decrease in internode length and increase in radial expansion. This type of gradual response is the most common thigmomorphogenic response observed in plants^4^. Hence, although there is a strong correlation between the degree of longitudinal strain experienced and the extent of the thigmomorphogenic response^22^, reduced internode elongation and increased plant compactness are known primary thigmomorphogenic plant responses among many species^23^.

**Figure 5:**
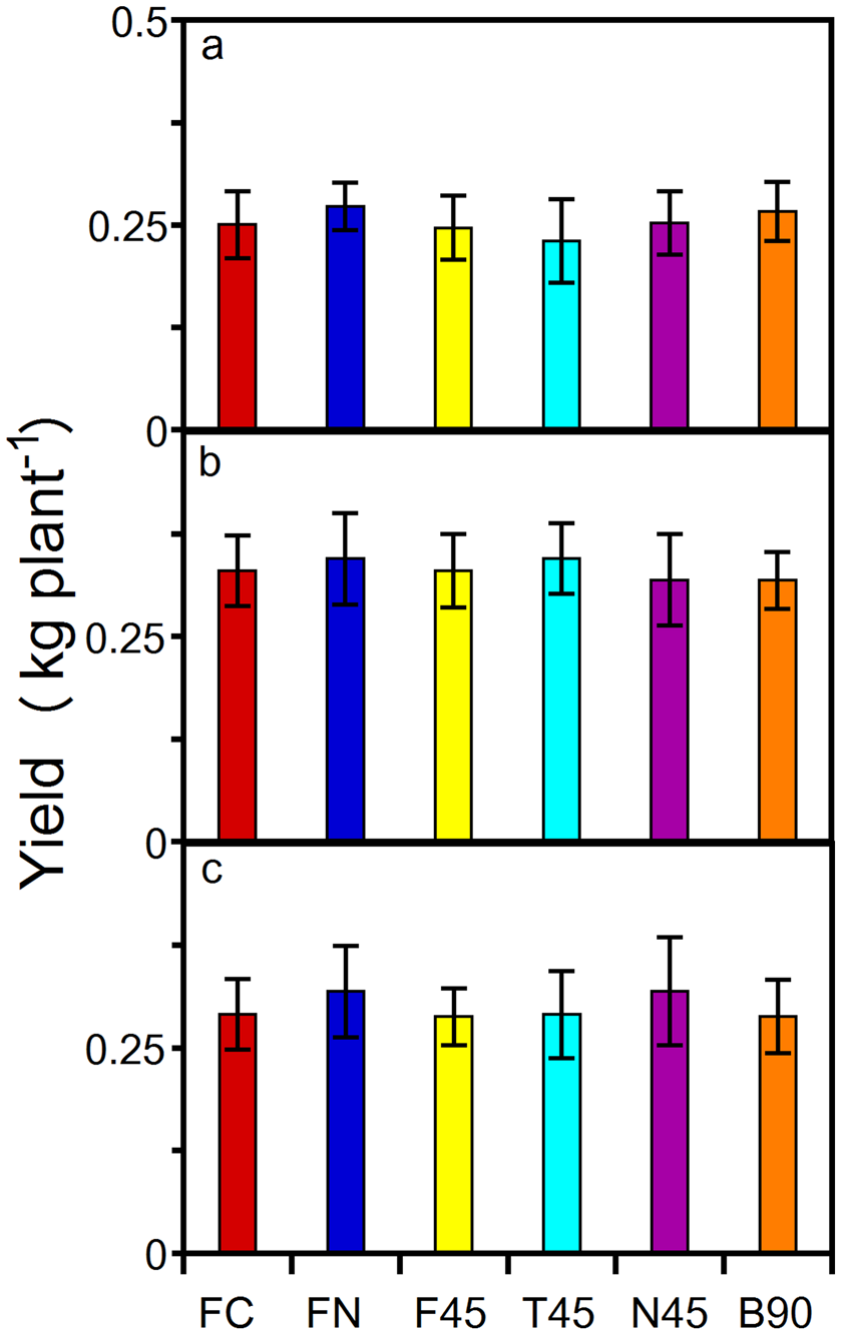
The hop mean dry cone yield (node 20–40; kg) across treatments for (**a**), cultivar ‘Cascade’. (**b**), cultivar ‘Cashmere’, and (**c**), cultivar ‘Centennial’. Control free to climb at 90° (FC), free to climb a strand of 90° netting (FN), free to climb twine at 45° (F45), touch on twine at 45° (T45), touch on a single strand of trellis netting at 45° slope (N45), and 90° internode bending on netting at a 45° mean slope (B90). Means and standard deviation of six replicates (n = 6). Yield means were not statistically different from each other (*P* > 0.32).

No significant differences were observed among internode lengths among the FC, FN, and F45 treatments (Table 2). This is one indication that the 45° slope in the untouched F45 treatment did not impart a gravitropic effect on internode length and node development. Secondly, when a plant stem is artificially bent, the plant’s orientation to the gravity field is changed as opposed to when a plant is tilted and bends under its own weight^24^. The resulting change in the gravity field normally makes it hard to disentangle thigmomorphogenesis from gravitropism. Hops, however, lack a self-supporting stem and thus rely on an independent physical support structure for vertical ascent. The independent support structure/trellis prevents the bine from experiencing gravity when tilted or bent at an angle because the structure counteracts the effects of gravity (e.g. F45 and B90). Thus, this study attempted to remove the gravity component as a potential culprit of decreased internode length or increased width, leaving only thigmomorphogenisis as the primary stimuli. In so doing, it was quantitatively apparent that the yields did not suffer with the introduction of a 45° bine slope or multiple mechanically induced internode bends (Fig. 5). To illustrate, Fig. 3 shows a subsection of cultivar ‘Centennial’ canopy with bent internodes supported by netting at a mean slope of 45°, the quantity of cones produced was copious (Fig. 3). Figure 4 illustrates the non-bent versus bent node elongation zone, showing a shorter internode that results in a higher density of cones along the length of the bine.

There was not a significant difference between treatments T45 and N45 (two tailed t-test; P > 0.14) where on average the intraspecific internode difference between T45 and N45 was 1.2%, 2.3%, and 5.6% for ‘Cashmere’, ‘Cascade’ and ‘Centennial’ respectively. Bending a stem introduces a local strain^14,22,25,26^. It is the local strain, not force to which plants respond, signaling a modification to the active elongation zone (local perception)^14^. Although this would indicate that the touching and bending treatments solely have a local influence on hop node elongation, more research is needed to substantiate that assertion.

In nature, wind exerts force and a resulting strain on plants during shoot elongation^27^. This study minimized the added effects of widespread wind strain in a controlled environment in an attempt to isolate long distance strain from that perceived at an internode. Although the elongation zone of a hop bine autonomously coils in search of vertical support, human touch and applied strain within the youngest main bine apical tissue (the youngest ~ 0.5 m of apical bine) resulted in local internode biomass reallocation from length to width. Therefore, new growth of hop bines appears to respond to both components of mechanical perturbation (touch and strain). This finding illustrates that the new growth at the apical end of the hop bine does not desensitize from repeated touching and bending, at least not when the stimulus occurs once per 24 h. Moreover, it is likely that this zone of growth would be the most affected by the stimuli e.g.^1,28^.

With respect to cone yield, the FT, FN, F45, T45, N45, and B90 treatments for cultivars ‘Cascade’, ‘Centennial’, and ‘Cashmere’ were not different (Fig. 5; two-way ANOVA; *P* > 0.32). Moreover, the flower yield of treatments T45, N45, and B90 was not significantly different than FC, FN, and F45 (Fig. 5) nor was the mean flower dry weight affected by touch or bending treatments. Regardless of similar yields among treatments, the benchmark for comparison of hops grown under any type of controlled-environment conditions are the α and β brewing characteristics as compared to field-grown hops. The analysis of α and β acids present in hop flowers describes hop cone chemical quality for brewers. The acid concentrations determine market value^29^. The percentages of α and β acids in the cone are used in the brewing process to estimate the bitterness profile in beer. Comparing measured values in this study with field grown literature values showed that α and β acids were not adversely impacted by controlled-environment conditions nor were they impacted by the thigmomorphogenic stimuli (Table 3). Furthermore, the concentrations of α and β acids grown under controlled-environment conditions with mechanical perturbation were within the typical cultivars’ field-grown α and β-acid range (Table 3)^30–35^.

**Table 3:** Observed alpha and beta-bitter acids of hop cultivars across treatments. Control free to climb at 90° (FC), free to climb a strand of 90° netting (FN), free to climb twine at 45° slope (F45), touch on twine at 45° slope (T45), touch on a single strand of trellis netting at 45° slope (N45), and 6) 90° internode bending on netting at a 45° mean slope (B90). Observed means of n = 4 100 g cone subsamples per cultivar per treatment ± standard deviation. Acid values are cone dry weight percentages. Alpha and beta-bitter acids means among treatments were not statistically different from each other (*P* > 0.17).

Cultivar	FC	FN	F45	T45	N45	B90
*Alpha*						
*‘Cascade’*	7.1 ± 0.6	7.4 ± 0.5	6.9 ± 0.3	7.5 ± 0.7	8.0 ± 0.8	7.3 ± 0.7
*‘Cashmere’*	7.9 ± 0.4	7.3 ± 0.6	6.4 ± 1.1	6.8 ± 0.6	7.4 ± 1.0	7.6 ± 0.8
*‘Centennial’*	9.6 ± 0.8	8.9 ± 0.5	9.2 ± 0.8	9.3 ± 1.1	9.0 ± 0.9	9.4 ± 0.6
*Beta*						
*‘Cascade’*	6.5 ± 1.1	6.1 ± 0.9	6.8 ± 0.6	7.0 ± 1.4	6.9 ± 0.9	6.4 ± 1.3
*‘Cashmere’*	6.4 ± 0.9	6.2 ± 1.1	5.9 ± 1.3	6.8 ± 0.5	5.7 ± 1.2	6.1 ± 0.8
*‘Centennial’*	3.2 ± 0.4	3.6 ± 0.6	3.7 ± 0.3	4.0 ± 0.9	3.5 ± 0.5	3.9 ± 0.6

Future experiments should look at the combination of mechanical perturbation with an artificially extended vegetative cycle because hop bine node quantity is directly related to total bine yield. Our controlled environment space constraints did not permit variation in the quantity of nodes developed among treatments prior to photoperiod induced flower initiation. However, hop bines grew to 22.5 m when artificially extending the vegetative phase via photoperiod extension^36^. Given that hop bines in the vegetative cycle under controlled environment conditions produce a new visible node approximately every 1–2 d^−1^ (depending on variety), it could be economically valuable to extend the vegetative portion of the crop cycle in order to increase overall crop yield. The reasons are hop production cycles necessitate a protracted juvenile phase during which they are incapable of flowering^7^. This stipulation not only results in bines that are of significant length prior to reaching maturity, it reduces their productivity to only the adult phase nodes that are present in a confined space i.e. nodes > 12–25. Thus, amassing many fertile nodes per vertical distance within a high sidewall greenhouse (e.g. ≥ 6 m) would be one viable means to increase the yield potential of hop in controlled environment production as long as plant resources did not become limiting. What’s more the 15.25 cm rise over run staircase created by the B90 internode bending treatment would allow for approximately double the bine length from the container to the top of a high sidewall greenhouse as compared to a vertically trellised bine (an additional direct step toward increasing node quantity per unit vertical production area). Secondly, the time and resource investment in overcoming the hop cultivar specific 11–24 infertile juvenile phase adds approximately three weeks to a single hop crop cycle e.g.^11,36^. Thus, it would be more time and space efficient to grow fewer crop cycles per annum that contain larger amounts of fertile nodes within a cycle as compared to additional cycles that contain the unfertile juvenile phase.

In conclusion, repeated touch and/or bine bending within the active elongation zone of hop bines resulted in shortened internode length with higher cone production per given area. Mechanical stimuli did not reduce cone yield or flower quality. The results demonstrate that successive local internode strain can aid the control of internode elongation. Moreover, the study provides evidence that thigmomorphogenic cues can be used as a management tool to increase bine compactness and increase node density per unit area. This finding is especially important for growth control when production space is limiting and/or of high-value (e.g. greenhouse production)^1^. Hence, mechanical perturbation was an effective non-chemical means to control hop internode length. Nonetheless, models aimed at predicting internode length of hop bines in response to strain should still take into account a cultivar parameter. The results are practical on a commercial scale because the methods of touch and bending used in this study are easy to apply with minimal investment in labor, have a short time interval of application (approximately 5–10 s^−1^ per bine per 24 h), and the application duration is relatively short ~ 30 days out of the 90–120 day crop cycle, making this a practical endeavor when one considers that high value vine crops are already repeatedly handled by humans throughout their production cycle (e.g. viticulture grape and controlled environment cucumber production).

## Plant material

Permission to collect and grow *Humulus lupulus* plants were obtained. The handling of *Humulus lupulus* plants were carried out in accordance with relevant guidelines and regulations.

## Supplementary Information

Supplementary Information

## Data Availability

The datasets generated during and/or analyzed during the current study are available from the corresponding author on reasonable request.
