# Supplementary material for: Internode elongation and strobili production of Humulus lupulus cultivars in response to local strain sensing
Source: Sci Rep. 2021 Apr 27;11:9017. doi: 10.1038/s41598-021-88720-8 (PMC8079374; doi:10.1038/s41598-021-88720-8)
Supplement: Supplementary file 1 — Supplementary Information [file 41598_2021_88720_MOESM1_ESM.pdf]

Supplementary information

Internode elongation and strobili production of *Humulus lupulus* cultivars in response to local strain sensing

William L. Bauerle[1*]

[1]Department of Horticulture and Landscape Architecture,

Colorado State University, Fort Collins, CO 80523

*Corresponding author: William L. Bauerle

Correspondence email: bauerle@colostate.edu

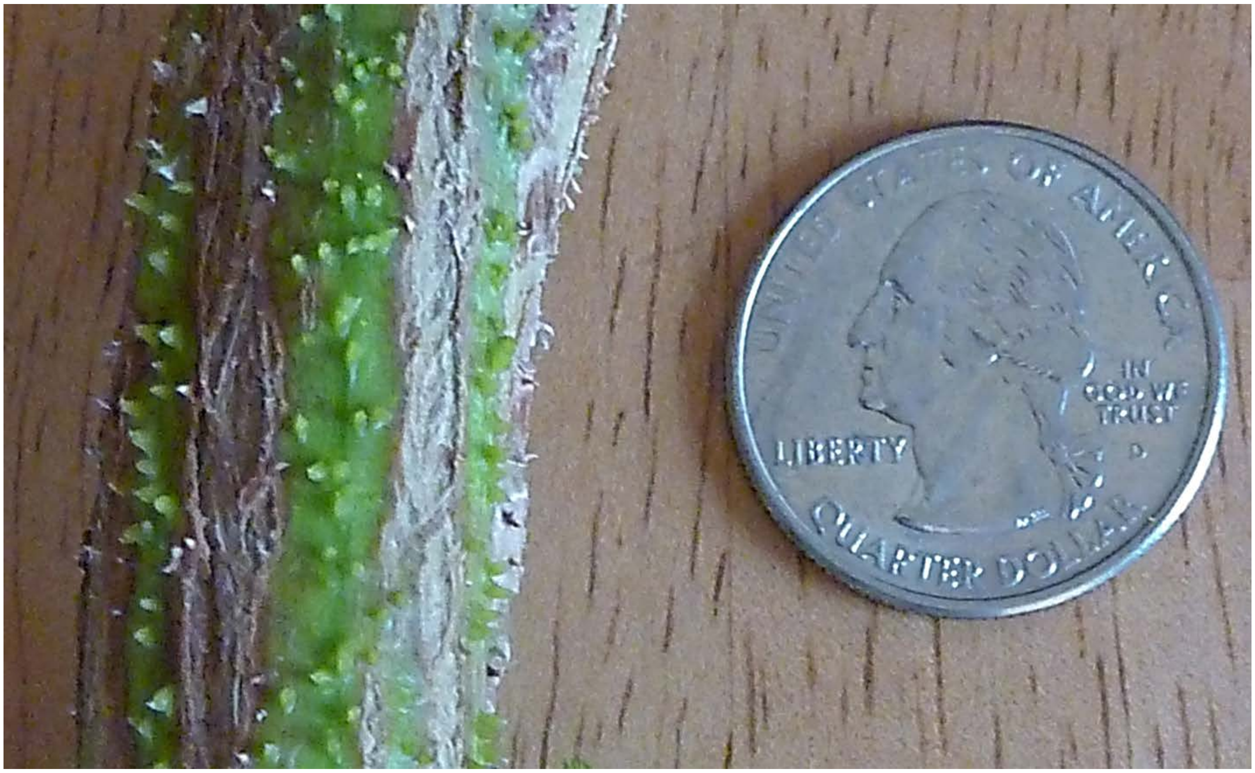

Supplementary Figure 1. The naturally formed silica hairs on the main bine that adhere the stem to the trellis support structure. The USA $0.25 cent piece (right) illustrates the relative size of a 100 d old greenhouse grown bine with silica hairs.

Supplementary Table 1. List of hop cultivars used in this study, their relative field-grown harvest time, brewing use, and year of public release. Harvest time: E= early, M = medium. NA = not available.

Cultivar	Harvest time	Brewing use	Year of release
'Cascade'	M	Dual purpose	1972
'Cashmere'	M	Aroma	2013
'Centennial'	E - M	Dual purpose	1991
